# Supplementary material for: Substrate Stiffness Modulates Renal Progenitor Cell Properties via a ROCK-Mediated Mechanotransduction Mechanism
Source: Cells. 2019 Dec 3;8(12):1561. doi: 10.3390/cells8121561 (PMC6953094; doi:10.3390/cells8121561)
Supplement: Supplementary file 1 [file cells-08-01561-s001.pdf]

	1 Vs 0.2	2 vs 1	4 vs 0.2	4 vs 0.5	4 vs 1	4 vs 2	8 vs 0.2	8 vs 0.5	8 vs 1	8 vs 2	8 vs 4	12 vs 0.2	12 vs 0.5	12 vs 1	12 vs 2	12 vs 4	25 Vs 0.2	25 Vs 0.5	25 vs 1	25 vs 2	25 vs 4	50 Vs 0.2	50 Vs 0.5	50 vs 1	50 vs 2	50 vs 4	50 vs 25
**circularity**	1	0	1	1	0	1	1	1	1	1	0	1	1	0	1	0	1	1	1	1	0	1	1	0	0	0	1
**spreading**	0	0	1	1	1	1	1	1	1	1	1	1	1	1	1	1	1	1	1	1	1	1	1	1	1	1	0
**Axis ratio**	1	1	1	1	0	1	1	1	0	1	0	1	1	0	1	0	1	1	0	1	0	1	0	0	1	0	0

Table 1. Means comparison obtained after ANOVA with post-hoc Tukey test.

1 indicates that the difference of the means is significant at the 0.05 level.

0 indicates that the difference of the means is not significant at the 0.05 level
